# Immediate Sequential Bilateral Cataract Surgery: Opinions among Refractive Surgeons in the United States and a Comparative Analysis with European Consultants

**DOI:** 10.1155/2022/8310921

**Published:** 2022-09-05

**Authors:** Sloan W. Rush, Andres E. Guerrero Criado, Guy M. Kezirian, Daniel Durrie

**Affiliations:** ^1^Panhandle Eye Group, 7400 Fleming, Amarillo 79106, TX, USA; ^2^Texas Tech University Health Science Center, 1400 S. Coulter, Amarillo 79106, TX, USA; ^3^SurgiVision Consultants, 28071 N. 90^th^ Way, Scottsdale 85262, AZ, USA; ^4^Durrie Vision, 8300 College Blvd Suite 201, Overland Park 66210, KS, USA

## Abstract

**Purpose:**

To analyze the perspectives of practicing refractive surgeons regarding the implementation of Immediate Sequential Bilateral Cataract Surgery (ISBCS) in the United States (US) and to compare their perspectives with those of European colleagues. *Setting*. Online refractive surgery forum.

**Design:**

A survey-based questionnaire.

**Methods:**

An electronic survey was emailed to all surgeon members of the Refractive Surgery Alliance (RSA) in the US. Participants were prompted to score their impressions regarding various aspects regarding ISBCS. Responses were compared to published reports conducted among European surgeons.

**Results:**

The electronic link to the survey was emailed to US-based surgeon members of the RSA, where 107 participated (44.6%). Twenty-seven (25.2%) reported that they currently perform ISBCS. Twenty-three (22.5%) of the respondents indicated they felt ISCBCS should be offered as a standard of care for routine cataract surgery. For surgeons that do not perform ISBCS, the most important factors were related to medicolegal issues and decreased reimbursement, whereas evidence of effectiveness and complications related to ISBCS were less important. Compared to practitioners abroad, 67.2% of European ophthalmic surgeons, compared to 25.2% of US surgeons, perform ISBCS (*p* < 0.0001).

**Conclusions:**

While US refractive surgeons often perform bilateral corneal procedures, many significant barriers exist to the widespread adoption of ISBCS. Concerns reported by US surgeons mirror those reported by surgeons in Europe. The majority of the US refractive surgeons in this survey indicate that ISBCS should not be the standard of care in routine cases, with the prevailing reason being concerns about decreased physician reimbursement and potential medicolegal issues, not safety.

## 1. Introduction

Cataract surgery is the most common outpatient surgical procedure performed in the US. It is estimated that greater than 24 million Americans have cataracts in one or both eyes, and this number is expected to double over the next 30 years [1]. Currently, an estimated 3.5 million patients have cataract surgery each year in the US [2] with the vast majority of them being performed in the context of delayed sequential bilateral cataract surgery (DSBCS) [3]. DSBCS has been the standard of care since the inception of cataract surgery. But as early as 2012, some investigators have argued that immediate sequential bilateral cataract surgery (ISBCS) should be the standard of care [4], though there has always existed a strong opposing viewpoint and resistance to change [5].

Numerous reports in both the US and abroad have demonstrated the safety and efficacy of ISBCS [6, 7]. Safety concerns include risk for bilateral endophthalmitis, bilateral toxic anterior segment syndrome (TASS), and inability to refine lens implant calculations for the fellow eye [8, 9], but these concerns have largely not been supported by findings in the literature when proper protocols are used [10–13]. Patient benefits from ISBCS include more rapid binocular vision recovery, a decreased number of office visits, and a decreased amount of travel time, whereas the healthcare system benefits from decreased costs [14–16]. Even still, some experts have cited the lack of strong enough evidence as being a significant barrier for the implementation of ISBCS as the standard of care [17, 18].

Outside of the US, investigators have determined many nonfinancial reasons as to why widespread adoption of ISBCS has been limited. These practitioners have cited risks of endophthalmitis, medicolegal issues, incorrect intraocular lens power calculation, and lack of board/society approval [19–21]. Many of these same reasons are applicable to US surgeons as well, but the financial constraints may be unique to the healthcare system in the US. Increasing costs associated with the delivery of healthcare in the US are driving the demand to find more efficient ways to save costs. Data have shown that the increasing financial strains associated with decreased physician reimbursement from Medicare are leading to more billing [22]. In this study, we evaluate the opinions among US surgeons with regards to both the safety of ISBCS and the financial limitations present in the evolving US healthcare system and contrast them with those of European surgeons.

## 2. Methods

The SRS Institutional Review Board (IRB00009122) approved this survey-based questionnaire that analyzed physician responses and their viewpoints concerning ISBCS. All elements of the study observed the principles of the Declaration of Helsinki and were with carried out with regards to human research standards and regulations.

### 2.1. Questionnaire Design and Distribution

The questionnaire was developed to mirror surveys that have been performed among European consultants in previously published studies to allow for a more accurate comparative analysis [20]. A link and QR code to the electronic survey was sent by e-mail to all surgeon members of the Refractive Surgery Alliance (RSA) practicing medicine in the US. The survey was conducted using cloud-based software from Research Electronic Data Capture (REDCap). Study participants were prompted to score their impressions on a Likert-based scale regarding various aspects regarding ISBCS. The replies were collected on a scaled score according to the respondent's rated level of importance. The responses were anonymously collected from the online software and maintained as confidential.

### 2.2. Data Collection and Statistical Analysis

REDCap was used to create pie chart and bar graph computations of the survey responses. JMP 11 software from the SAS Institute (Cary, NC, USA) was used to generate percentages, means, and standard deviations. Contingency analysis with likelihood ratios was used to compare outcomes in this study and a similar study conducted among European consultants [20]. The results were considered statistically significant at the alpha <0.05 level. Incomplete surveys were excluded from the analysis.

## 3. Results

A total of 107 of 240 (44.6%) US-based surgeon members of the RSA completed the survey and were included in the analysis. Of the respondents, 27 (25.2%) reported they currently perform ISBCS, 75 surgeons (70.1%) indicated they do not perform ISBCS, and 5 surgeons (4.7%) indicated they have discontinued performing ISBCS. The characteristics of the survey respondents indicated a wide range of diversity without any notable geographic or length of practice predilection (Table 1).

### 3.1. Practitioners of ISBCS

There were 29.9% (*n* = 32) of the survey respondents that have performed ISBCS in routine cases. The factors considered most important among this cohort are that the surgeon has a low complication rate (75.0%) as well as multiple matters related to infection risk, including surgeons/nurses regloving and regowning between eyes (87.5%), the patient's being at low risk for infection (78.1%), and the operating facility's infection record (71.9%). Several factors unrelated to safety were regarded as less important, which included time savings and convenience for the patient (62.5%), reimbursement issues (46.9%), and cost savings for the healthcare system (34.4%). The least important factor was having a second surgeon and scrub nurse for the second eye (0.0%). The summary of these findings is displayed in Table 2.

### 3.2. Nonpractitioners of ISBCS

There were 70.1% (*n* = 107) of the survey respondents that do not perform ISBCS in routine cases.  Table 3 shows the distribution of responses among these surgeons. The concerns regarded as most important include medicolegal issues (58.7%), reimbursement issues (54.7%), and the risk of endophthalmitis (46.7%). The least important worries were lack of training (1.3%), lack of evidence regarding ISBCS efficacy (5.3%), and lack of available support staff (8.0%).

### 3.3. Future Outlook for ISBCS

Medicolegal/indemnity insurance approval was regarded as the most important circumstance for all surgeons to routinely practice ISBCS as the standard of care (65.4% of respondents). Other less important circumstances included improved evidence of safety and effectiveness (41.1%), specialist society or academy approval (38.3%), availability of prepacked right eye/left eye instruments (32.7%), and improved availability of intracameral antibiotics (29.9%). Thirteen (12.1%) respondents stated that they would not perform ISBCS under any circumstances. These findings are given in Table 4.

### 3.4. Comparative Analysis

Compared to practitioners abroad, [20] 67.2% of European ophthalmic surgeons compared to 25.2% of the US surgeons perform ISBCS (*p* < 0.0001). Among those that do not perform ISBCS, two of the most important factors among both US and European surgeons are the risk of endophthalmitis and medicolegal issues should ISBCS go wrong. With respect to risk for endophthalmitis, 69.0% of European surgeons compared to 46.7% of US surgeons rate this factor as a very important reason for not performing ISBCS (*p*=0.006) and with respect to medicolegal issues, 57.8% of European surgeons compared to 58.7% of the US surgeons rate this factor as a very important reason for not performing ISBCS (*p*=0.91).

## 4. Discussion

This survey has explored the benefits, disadvantages, and barriers to adopting ISBCS as the standard of care practice among US surgeons. The majority of surgeons do not believe that ISBCS should be the standard of care for routine cataract surgery at this particular point in time. But their attitudes toward ISBCS are still favorable under the right circumstances, and ISBCS may become more accepted over time, as only 12.1% state that they would not ever consider ISBCS. The primary limiting factors are related to medicolegal and reimbursement issues, two reasons unrelated to patient care. Approximately half of both ISBCS practitioners and nonpractitioners alike regarded reimbursement issues as very important, with no statistical significance between the two groups (*p*=0.46). This finding suggests that reimbursement is an obstacle even for those that routinely perform ISBCS and that they may be suffering financial consequences as a result of practicing ISBCS.

There are a variety of practice settings that may influence reimbursement in the US. Practices that perform predominantly elective refractive lens exchange procedures or do not accept medical insurance may have no financial consequences. Other settings that include academic medical practice, the Veteran's Affairs healthcare system, and health management organization networks [8] may impose minimal financial barriers for the surgeon. By contrast, practices that rely heavily on reimbursement from third-party payers, including private insurance and Medicare, may experience significant revenue loss when adopting ISBCS [14]. Both medicolegal and financial aspects in the US are likely to evolve over time, which may positively influence the attitudes of surgeons regarding ISBCS. Physicians can inform policy-makers regarding the cost savings of ISBCS for the healthcare system while noting that a large decrease in physician reimbursement will inhibit its practice and widespread adoption of ISBCS will mitigate many concerns related to medicolegal issues.

The largest barrier to routine ISBCS implementation that relates directly to patient care is a concern for endophthalmitis. Arshinoff et al. have developed safety guidelines when performing ISBCS [23]. When using published protocols and treating both eyes as separate surgical events, the likelihood of bilateral simultaneous endophthalmitis is exceedingly low. Reported cases have been the consequence of major breachs in sterile technique or otherwise substandard surgical techniques [13]. Interestingly, US surgeons have less concern regarding the importance of endophthalmitis than their European counterparts (*p*=0.006), yet European surgeons are still greater than 2.5 times more likely to perform ISBCS. This disparity could possibly be explained by the decreased incidence of endophthalmitis reported in the US compared with Europe. [24, 25] Though commonly used in practice by many US surgeons, there is still a lack of an FDA-approved intracameral antibiotic for infection prevention during cataract surgery. This conundrum persists due to the high costs associated with the FDA-approval process, despite overwhelming evidence of its effectiveness. [26].

It is revealing that only 5.3% of surgeons not practicing ISBCS cite a lack of evidence for the effectiveness of ISBCS as very important. This indicates that more peer-reviewed, published evidence may not have that large of an impact on the attitudes or beliefs about ISBCS among US surgeons. It would be of interest to see how removing the financial and medicolegal obstacles may influence surgeon attitudes about ISBCS. Though not the only consideration, patient care should be prioritized above all else. Also, this survey does not capture any input from the patient, but it can be assumed that there would be time and cost savings as well as convenience for the patient in the setting of ISBCS. [27, 28] The surgeons performing ISBCS in this study indicated some concern about these issues.

Additional weaknesses of this study consist of its inclusion of only physicians that identify as refractive surgeons and may routinely be accustomed to practicing bilateral surgery (as in the setting of corneal refractive procedures), the relatively small number of survey respondents, and the potential for bias in an uncontrolled and unvalidated, Likert-based survey design. Additional research is warranted to further investigate the barriers and limitations that prevent more widespread use of ISBCS in the US.

## Figures and Tables

**Table 1:** Characteristics of survey respondents.

Characteristics (*n* = 107)	
Region of practice in the United States	Northeast = 13.1 (14)
	Midwest = 29.0 (31)
	South = 32.7 (35)
	West = 24.3 (26)
	Other = 0.9 (1)
Length of practice	<5 years = 15.9 (17)
	5–10 years = 15.0 (16)
	11–25 years = 33.6 (36)
	>25 years = 35.5 (38)
Access to an ambulatory surgery center	Yes = 86.9 (93)
	No = 13.1 (14)
Practice office-based surgery	Yes = 22.4 (24)
	No = 77.6 (83)
Annual number of cataract surgeries performed	<100 = 10.3 (11)
	101–500 = 30.8 (33)
	501–1,000 = 30.8 (33)
	1,001–2,000 = 19.6 (21)
	>2,000 = 8.4 (9)

Values are given in % and (*n*).

**Table 2:** Aspects to consider among surgeons who have performed ISBCS.

Aspects for consideration (*n* = 32)	Very important	Important	Quite important	Not important
Surgeon and scrub nurse regown and reglove before surgery on the second eye	87.5 (28)	9.4 (3)	3.1 (1)	0 (0)
Patient has no additional risk factors for endophthalmitis	78.1 (25)	12.5 (4)	6.3 (2)	3.1 (1)
Facility's infection record	71.9 (23)	18.8 (6)	0 (0)	9.4 (3)
Have a second surgeon and scrub nurse for the second eye	0 (0)	0 (0)	3.1 (1)	96.9 (31)
More cost-effective for the health system	34.4 (11)	28.1 (9)	18.8 (6)	18.8 (6)
Better visual outcome for the patient	37.5 (12)	40.6 (13)	3.1 (1)	18.8 (6)
More convenient for a patient with faster rehabilitation	62.5 (20)	25.0 (8)	3.1 (1)	9.4 (3)
Reduced visits for patient time savings	62.5 (20)	25.0 (8)	6.3 (2)	6.3 (2)
Saves time in clinic and operating room	46.9 (15)	34.4 (11)	3.1 (1)	15.6 (5)
Exclusion of high-risk eyes	65.6 (21)	25.0 (8)	3.1 (1)	6.3 (2)
Surgeon has a low complication rate	75.0 (24)	18.8 (6)	3.1 (1)	3.1 (1)
Instruments have gone through different sterilization cycles	65.6 (21)	21.9 (7)	0 (0)	12.5 (4)
Medicine, solutions, and cannulas come from different manufacturers or have different batch numbers	21.9 (7)	43.8 (14)	6.3 (2)	28.1 (9)
Postoperative day 1 review by an ophthalmologist	37.5 (12)	28.1 (9)	15.6 (5)	18.8 (6)
Reimbursement issues	46.9 (15)	40.6 (13)	6.3 (2)	6.3 (2)

Values are given in % and (*n*).

**Table 3:** Concerns among surgeons about not performing ISBCS.

Concerns (*n* = 75)	Very important	Important	Quite important	Not important
Risk for endophthalmitis	46.7 (35)	20.0 (15)	18.7 (14)	14.7 (11)
Medicolegal issues should ISBCS go wrong	58.7 (44)	26.7 (20)	6.7 (5)	8.0 (6)
Risk for incorrect IOL power calculation	21.3 (16)	25.3 (19)	25.3 (19)	28.0 (21)
Insufficient facilities or support staff	8.0 (6)	6.7 (5)	13.3 (10)	72.0 (54)
Lack of training	1.3 (1)	13.3 (10)	8.0 (6)	77.3 (58)
No evidence of effectiveness	5.3 (4)	10.7 (8)	18.7 (14)	65.3 (49)
Risk for postoperative cystoid macular edema	13.3 (10)	20.0 (15)	18.7 (14)	48.0 (36)
Risk for retinal detachment	9.3 (7)	20.0 (15)	21.3 (16)	49.3 (37)
Reimbursement issues	54.7 (41)	17.3 (13)	13.3 (10)	14.7 (11)

Values are given in % and (*n*).

**Table 4:** Circumstances that will positively influence the decision for routinely performing ISBCS.

Circumstance (*n* = 107)	
Improved evidence of safety and effectiveness	41.1 (44)
Medicolegal/indemnity insurance approval	65.4 (70)
Specialist society or academy approval	38.3 (41)
Availability of prepacked right eye/left eye instruments	32.7 (35)
Improved availability of intracameral antibiotics	29.9 (32)
Other	19.6 (21)
I would not consider bilateral cataract surgery under any circumstances	12.1 (13)

Values are given in % and (*n*).

## Data Availability

The datasets used and/or analyzed during the current study are available from the corresponding author upon reasonable request.

## Abbreviations

ISBCS: Immediate sequential bilateral cataract surgery
DSBCS: Delayed sequential bilateral cataract surgery
RSA: Refractive surgery alliance.

## Additional Points

*Synopsis*. The majority of surveyed surgeons indicate that ISBCS should not be the standard of care, with the prevailing reason being concerns about decreased reimbursement and potential medicolegal issues, not safety.
